# HDAC inhibitor MS275 reprograms metabolism to induce differentiation and suppress proliferation in hepatocellular carcinoma

**DOI:** 10.3389/fimmu.2025.1623211

**Published:** 2025-09-16

**Authors:** Jingjie Li, Cheng Hu, Yuyu Ye, Song Wei, Wenbo Zhu, Jiankai Liang, Jing Cai, Yuan Lin, Liang Peng, Guangmei Yan, Ying Liu

**Affiliations:** ^1^ Reproductive Medicine Research Center, The Sixth Affiliated Hospital, Sun Yat-sen University, Guangzhou, China; ^2^ Department of Urology, The Third Affiliated Hospital of Sun Yat-sen University, Guangzhou, China; ^3^ Department of Infectious Diseases, The Third Affiliated Hospital of Sun Yat-sen University, Guangzhou, Guangdong, China; ^4^ Department of Pharmacology, Sun Yat-sen University, Guangzhou, China

**Keywords:** HDAC inhibitor, MS275, hepatocellular carcinoma, oxidative phosphorylation, glycolysis, metabolic reprogramming, PKM1, ROS

## Abstract

**Background:**

Histone deacetylase (HDAC) inhibitors have shown therapeutic promise in various cancers, including hepatocellular carcinoma (HCC), due to their ability to regulate cell proliferation, differentiation, and apoptosis. However, their role in metabolic reprogramming and differentiation therapy in HCC remains underexplored.

**Methods:**

This study investigated the effects of the HDAC inhibitor MS275 on HCC cells *in vitro* and *in vivo*. Cell viability, differentiation marker expression, cell cycle distribution, metabolic activity, and reactive oxygen species (ROS) production were evaluated using CCK-8 assays, qRT-PCR, flow cytometry, Seahorse metabolic analysis, and western blotting. A xenograft mouse model was used to validate *in vivo* efficacy.

**Results:**

MS275 significantly suppressed HCC cell proliferation by inducing G0/G1 phase arrest without triggering apoptosis. MS275 also upregulated hepatocyte-specific markers (GLUL, HNF1A, HNF3A), indicating that it promoted differentiation. Mechanistically, MS275 reprogrammed cellular metabolism by enhancing oxidative phosphorylation and reducing glycolysis, accompanied by increased expression of the metabolic enzyme PKM1. This metabolic shift led to elevated ROS production, which was essential for MS275-induced differentiation. Knockdown of PKM1 abolished both the differentiation and anti-proliferative effects. *In vivo*, MS275 significantly reduced tumor growth and promoted differentiation without systemic toxicity.

**Conclusion:**

MS275 suppresses HCC cell proliferation and induces hepatocyte-like differentiation through PKM1-mediated metabolic reprogramming and ROS signaling. These findings support the potential of MS275 as a differentiation-based therapeutic strategy for HCC.

## Introduction

Hepatocellular carcinoma (HCC) is one of the most common and aggressive liver malignancies and ranks as the third leading cause of cancer-related mortality worldwide (1). Its incidence has been steadily increasing, particularly in regions with a high prevalence of chronic liver disease, such as hepatitis B and C infections, alcoholic liver disease, and non-alcoholic steatohepatitis (NASH) (2). Despite advances in diagnostic and therapeutic approaches, the prognosis for HCC remains poor, with a five-year survival rate below 20% in most populations (3). High rates of tumor recurrence and metastasis after curative treatments such as surgical resection or liver transplantation significantly contribute to poor prognosis (4). HCC is characterized by aggressive cell proliferation, impaired differentiation, and resistance to apoptosis, all of which contribute to tumor progression and therapeutic resistance (5–7). Normal hepatocytes undergo a complex differentiation process, developing into specialized cells with specific functions. In HCC, this differentiation process is impaired, leading to the development of a more aggressive and poorly differentiated tumor phenotype (1). Moreover, HCC cells are often resistant to programmed cell death (apoptosis), enabling them to evade intrinsic and extrinsic death signals and persist under therapeutic stress (8). Given these challenges, there is an urgent need for novel therapeutic strategies.

Histone deacetylases (HDACs) are enzymes that remove acetyl groups from histone and non-histone proteins, leading to chromatin condensation and transcriptional repression (9). Aberrant HDAC activity is commonly observed in HCC patients. Specific HDACs, including HDAC1, HDAC2, HDAC4, HDAC7, and HDAC9, are strongly associated with HCC development and patient survival (10). By preventing this deacetylation, histone deacetylase inhibitors (HDACi) promote a more relaxed chromatin structure, which allows transcription of tumor suppressor genes and other regulatory factors involved in cell cycle arrest, apoptosis, and differentiation (11, 12). Among HDACis, suberoylanilide hydroxamic acid (SAHA, also known as vorinostat) is a pan-HDAC inhibitor that inhibits both Class I and Class II HDACs. In preclinical models, SAHA induces cell-cycle arrest and apoptosis in HepG2 and Huh-7 cells and has shown efficacy even in Lenvatinib-resistant HCC organoids (13, 14). Meanwhile, MS275 (entinostat) is a selective Class I HDAC inhibitor, with high specificity for HDAC1, HDAC2, and HDAC3. It has been reported to suppress tumor growth in tumor (15–17). MS275 induces differentiation at low doses and apoptosis at high doses in leukemia cells. Low concentrations promote p21-mediated growth arrest, while higher doses trigger ROS production, mitochondrial damage, and caspase-dependent cell death (18). In HCC, MS275 has demonstrated antitumor effects in both *in vitro* and *in vivo* models, including induction of apoptosis and potentially enhance the immune response against cancer cells (19, 20). Early-phase clinical trials have evaluated MS275 in solid tumors, establishing its maximum tolerated dose and characterizing its dose-limiting toxicities (21). Moreover, HDAC inhibitors also enhance the efficacy of first-line treatments like sorafenib, and can even re-sensitize resistant cells, highlighting their potential as combination agents in HCC treatment (22, 23).

Emerging evidence suggests that metabolic reprogramming is a hallmark of cancer and plays a crucial role in tumor progression (24). Unlike normal hepatocytes, which primarily rely on oxidative phosphorylation for energy production, HCC cells exhibit a shift toward aerobic glycolysis (the Warburg effect) to sustain rapid proliferation. Targeting this altered metabolism has gained interest as a potential therapeutic strategy for HCC (25, 26). In this study, we explored the antitumor mechanisms of the HDAC inhibitors MS275 (entinostat) and SAHA (vorinostat) in HCC cells. We observed that MS275 suppresses HCC cell growth by inducing G0/G1 cell cycle arrest without triggering apoptosis. MS275 also promotes hepatocyte-like differentiation, as shown by upregulation of *GLUL*, *HNF1A*, and *HNF3A*. Transcriptomic and metabolic profiling revealed that MS275 reprograms cellular metabolism by enhancing oxidative phosphorylation. This metabolic shift is mediated by upregulation of *PKM1* and is accompanied by increased mitochondrial ROS production, which is essential for MS275-induced differentiation and growth inhibition. *In vivo*, MS275 effectively reduced tumor growth in HCC xenografts without systemic toxicity, while promoting differentiation and reducing proliferation. These findings demonstrate that HDAC inhibition in HCC not only suppresses tumor growth but also promotes metabolic differentiation, offering a compelling therapeutic strategy that targets both the epigenetic landscape and altered metabolism of liver cancer.

MS275 (Entinostat) is a selective class I HDAC inhibitor known for its potent anti-cancer effects through cell cycle arrest, apoptosis induction, and differentiation promotion (16, 27, 28). However, the precise metabolic mechanisms by which MS275 suppresses HCC growth remain largely unexplored.

## Methods

### Cell culture and treatments

HCC cell lines HUH-7 and Hep-3B were purchased from the Cell bank of the Type Culture Collection of the Chinese Academy of Sciences (Shanghai, China) and ATCC (USA), respectively. They were cultured in DMEM supplemented with 10% fetal bovine serum (FBS), 1% penicillin/streptomycin at 37°C with 5% CO_2_. The human normal hepatocyte cell line L02 was cultured in DMEM supplemented with 20% FBS, antibiotics at 37°C under 5% CO_2_. L02 cells were obtained from Cell Bank of Type Culture Collection of Chinese Academy of Sciences. For treatment experiments, cells were seeded at a density of 2 × 10^5^ cells/well in 6-well (9.6 cm² surface area) or 1 × 10^4^ cells/well in 96-well plates (0.32 cm² surface area per well) and allowed to adhere overnight before drug exposure. HDACi, including MS275 (entinostat) and SAHA (vorinostat), were administered at indicated concentrations (typically ranging from 1 to 10 µM) for 24, 48, or 72 hours. To study the involvement of oxidative stress and metabolism, additional compounds were applied: N-acetylcysteine (NAC, 100 µM; Sigma-Aldrich, USA) was used to scavenge reactive oxygen species (ROS); Oligomycin A (0.5 nM; Sigma-Aldrich, USA) was used to inhibit ATP synthase and block oxidative phosphorylation; 2-deoxy-D-glucose (2-DG, 10 mM; Sigma-Aldrich, USA) was used to inhibit glycolysis; and Dichloroacetate (DCA, 10 mM; Sigma-Aldrich, USA) was used to enhance mitochondrial metabolism by inhibiting pyruvate dehydrogenase kinase. All compounds were prepared in DMSO or sterile PBS according to the manufacturer’s recommendations, and equivalent volumes of vehicle were added to control groups.

For gene silencing experiments, cells were transfected with small interfering RNA targeting *PKM1* (siPKM1) or non-targeting control siRNA (GenePharma, China) using Lipofectamine™ 3000 transfection reagent (Thermo Fisher Scientific, USA), following the manufacturer’s instructions. Briefly, siRNA-lipid complexes were prepared in Opti-MEM (Gibco, USA) and added to cells at 40–60% confluence. After 6 hours of incubation, the transfection medium was replaced with complete growth medium. Knockdown efficiency was confirmed by western blot. All experiments were independently repeated at least three times to ensure reproducibility and consistency.

### Cell viability assay

Cell viability was evaluated using the Cell Counting Kit-8 (CCK-8; C0038, Beyotime, China) according to the manufacturer’s instructions. Briefly, 5000 cells were seeded into 96-well plates (0.32 cm² surface area per well) and treated with the indicated compounds for the designated time periods. After treatment, 10 µL of CCK-8 reagent was added to each well, and the cells were incubated for 2 hours at 37°C in a humidified incubator. Absorbance was then measured at 450 nm using a microplate reader. All experiments were performed in triplicate, and results were expressed as a percentage of viable cells relative to control.

### RNA sequencing and data analysis

Total RNA was extracted using the TRIzol reagent according to the instructions. RNA quality were assessed using the Agilent 2100 Bioanalyzer. For each experimental condition, RNA was collected from three independent biological replicates. Sequencing libraries were prepared and sequenced on the Illumina NovaSeq 2000 platform to generate 150 bp paired-end reads.

Raw reads were quality-checked using FastQC, and adapter trimming was performed with Trim Galore. Clean reads were aligned to the reference genome GRCh38 using HISAT2. Aligned reads were then counted using featureCounts from the Subread package, generating a gene-level count matrix for downstream analysis. Differential expression analysis was conducted using the DESeq2 package (v1.36.0) in R. Genes with an adjusted p-value < 0.05 and |log_2_FoldChange| ≥ 1 were considered differentially expressed genes (DEGs).

Gene Ontology (GO) and Kyoto Encyclopedia of Genes and Genomes (KEGG) pathway enrichment analyses were performed using the clusterProfiler R package. To investigate glycolysis-related transcriptional changes, genes annotated under the GO term GO:0006096 (glycolytic process) were extracted. A heatmap of glycolysis-associated genes was generated using the pheatmap package in R to visualize expression patterns across samples.

### Quantitative real-time PCR

Total RNA was reverse-transcribed using the HiScript III RT SuperMix (Vazyme, China). qRT-PCR was performed using ChamQ SYBR qPCR Master Mix (Vazyme, China) on a QuantStudio 5 Real-Time PCR System (Applied Biosystems, USA). *ACTB* was used as the internal control. Relative gene expression levels were calculated using the 2^−ΔΔCt method. Primer sequences are listed in **Table 1**.

**Table 1:** Primer sequences used for qRT-PCR analysis.

Gene Symbol	Forward primer	Reverse primer
*GLUL*	CTCTCGCGGCCTAGCTTTAC	CGGAGTTCACAGAGTAGGCG
*HNF1A*	CCAGGTCTTCACCTCAGACAC	CTGCTGGAGGACACTGTGG
*HNF3A*	GGCTTCCTCTTCGCCCG	AGTAGGCCTCCTGCGTGT
*PKM1*	CGCATGCAGCACCTGATAGC	CAGAACTATCAAAGCTGCTGC
*PKM2*	CGCATGCAGCACCTGATTGC	GATTATGGCCCCACTGCAG
*hn RNPA1*	GCAATAGCAGGTGGAACCCT	GGAGCCATTCGCGCTATACT
*hn RNPA2*	TTGTCTAAAAGATCACCATCCCC	CAGCATTTTATTGGGGACAGCC
*PTB*	CTCACCAGCCTCAACGTCAA	TTATACCAGGTGCACCGAAGG
*ZFX*	TCCCTGAGCTGTGCTTTACG	CCCATCTTCATCCATGGCCT
*E2F1*	CGTGTCAGGACCTTCGTAGC	CCTCAGGGCACAGGAAAACA
*N-MYC*	CCTGCAGTCGGCGGGAGTGTT	AGCAGCTCGGCATCGGCTC
*STAT3*	GACATTCCCAAGGAGGAGGC	AGGTCGTTGGTGTCACACAG
*Nanog*	GAGGTCTCGTATTTGCTGCAT	ATTTCCTTCTCCACCCCAACC

### Apoptosis assay

HUH-7 cells (5 × 10³/well, 96-well plate) were treated with 10 μM MS275 or vehicle for 24, 48, and 72 h. After fixation (4% paraformaldehyde, 15 min) and permeabilization (0.1% Triton X-100, 5 min), cells were blocked with 5% normal goat serum (30 min) and incubated overnight at 4°C with rabbit anti-cleaved Caspase-3 (1:400; CST, 9661). Following three PBS washes, Alexa Fluor 488-conjugated goat anti-rabbit IgG (1:500; CST, 4412) was applied for 1 h at room temperature. Nuclei were counterstained with Hoechst 33342 (1 μg/mL, 5 min). Images were captured (≥5 fields/well) and analyzed with ImageJ. Apoptotic index was calculated as: (cleaved Caspase-3^+^ nuclei/total nuclei) × 100%.

### Seahorse metabolic analysis

Mitochondrial respiration and glycolytic activity were evaluated using the Seahorse XF Analyzer (Agilent Technologies, USA) to measure the oxygen consumption rate (OCR) and extracellular acidification rate (ECAR). HUH-7 and Hep-3B cells were seeded into XF96-well Seahorse microplates (0.106 cm² surface area per well) at a density of approximately 1.5 × 10^4^ cells per well and allowed to adhere overnight. Cells were then treated with MS275 or SAHA at the indicated concentrations and time points. On the day of the assay, the culture medium was replaced with Seahorse XF assay medium supplemented with glucose (10 mM), pyruvate (1 mM), and glutamine (2 mM), and cells were incubated in a CO_2_-free incubator at 37°C for 1 hour to allow for pH and temperature equilibration. For the Mitochondrial Stress Test, sequential injections of Oligomycin (1 µM), FCCP (1 µM), and a combination of Rotenone (0.5 µM) and Antimycin A (0.5 µM) were performed to assess basal respiration, ATP production, maximal respiratory capacity, and non-mitochondrial respiration. ECAR was simultaneously recorded to evaluate glycolytic activity. Each condition was tested in technical triplicates, and all experiments were independently repeated at least three times (n = 3). Data were normalized to total protein content or cell number and analyzed using Seahorse Wave software according to the manufacturer’s protocols.

### ATP and lactate measurement

Intracellular ATP levels were measured using the ATP Assay Kit (Beyotime, China) according to the manufacturer’s instructions. Briefly, HUH-7 and Hep-3B cells were seeded into 96-well plates at a density of 5,000 cells/well (0.32 cm² surface area) and treated with the indicated compounds. After treatment, cells were lysed, and the luminescent signal generated by the luciferase reaction was detected using a microplate luminometer, with signal intensity proportional to the ATP concentration. For lactate measurement, culture supernatants were collected, and lactate production was quantified using the Lactate Assay Kit (Bestbio, China). The assay was performed following the manufacturer’s protocol, and absorbance was measured at 570 nm using a microplate reader. All measurements were performed in triplicate, and results were normalized to cell number or total protein content where applicable.

### Flow cytometry for ROS measurement

ROS levels were measured using the fluorescent probe 2’,7’-dichlorodihydrofluorescein diacetate (DCFH-DA; Beyotime, China). HUH-7 and Hep-3B cells were seeded at a density of 2 × 10^5^ cells/well in 6-well plates (9.6 cm² surface area) and treated as described. After treatment, cells were washed with PBS and incubated with 10 µM DCFH-DA in serum-free medium at 37°C for 30 minutes in the dark. After incubation, cells were washed three times with PBS to remove excess dye and immediately analyzed using a BD FACSAria flow cytometer (BD Biosciences, USA). To specifically assess mitochondrial ROS, cells were stained with MitoSOX Red (M36007, Thermo Fisher Scientific, USA) in HBSS buffer at 37°C for 10 minutes in the dark according to the manufacturer’s protocol. After washing, cells were analyzed by flow cytometry under identical settings. All conditions were tested in technical triplicates, and each experiment was independently repeated at least three times (n = 3). Fluorescence intensity was quantified to evaluate total and mitochondrial ROS levels.

### EdU incorporation assay

Cell proliferation was assessed using an EdU incorporation assay. HUH-7 and Hep-3B cells were seeded at a density of 2 × 10^5^ cells/well in 6-well plates (9.6 cm² surface area) and treated with the indicated compounds. Following treatment, cells were incubated with 10 µM EdU (Sigma-Aldrich) for 2 hours, followed by chemical conjugation to 50 µM Auto 488 for 1 hour. Nuclei were stained with 2.5 µg/mL DAPI for 30 minutes before analysis. Samples were analyzed using a Gallios flow cytometer (Beckman Coulter). All conditions were analyzed in technical triplicates, and each experiment was independently repeated at least three times (n = 3).

### Cell cycle analysis

HUH-7 and Hep-3B cells were seeded at a density of 2 × 10^5^ cells/well in 6-well plates (9.6 cm² surface area) and treated as indicated. The protocol was improved based on the published method (29, 30).After treatment, cells were harvested by trypsinization, washed twice with PBS, and fixed in 4% paraformaldehyde at 4°C for 15 minutes, then incubated with 0.2% Triton X-100 in PBS (1 mL/well) for 3 min at room temperature to enable nuclear dye accessibility. Fixed cells were then washed with PBS and stained with 2.5 µg/mL DAPI for 30 minutes at room temperature in the dark. DNA content was measured using a Gallios flow cytometer (Beckman Coulter, USA), and cell cycle distribution (G_0_ /G_1_ , S, G_2_/M phases) was analyzed using FlowJo software (Tree Star Inc., USA). All conditions were analyzed in technical triplicates, and each experiment was independently repeated at least three times (n = 3).

### Western blot analysis

Cells or tissue were lysed in RIPA buffer (Beyotime, China) supplemented with protease and phosphatase inhibitor cocktails (Thermo Fisher Scientific, USA). Lysates were centrifuged at 12,000 × g for 15 minutes at 4°C to remove cellular debris. The supernatant was collected, and protein concentrations were determined using the BCA Protein Assay Kit (Thermo Fisher Scientific, USA) according to the manufacturer’s instructions. 20µg protein were separated by SDS-PAGE and transferred onto polyvinylidene fluoride (PVDF) membranes (Millipore, USA). Membranes were blocked with 5% non-fat milk in TBST for 1 hour at room temperature and then incubated overnight at 4°C with primary antibodies against GLUL (1:1000; abcam, ab64613), HNF1A (1:1000; Cell Signaling Technology[CST], 89670), HNF3A (1:1000; CST, 58613), PKM1 (1:1000; CST, 7067), PKM2 (1:1000; CST, 4053), PKM1/2 (1:1000; CST, 3190), GAPDH (1:1000; CST, 2146),Hexokinase I (1:1000; CST, 2024), Hexokinase II (1:1000; CST, 2867), PD (1:1000; Abcam, ab110335), PFKP(1:1000; CST, 3190),LDHA (1:1000; CST, 3582), and β-tubulin (1:1000; CST, 2146). After washing, membranes were incubated with appropriate HRP-conjugated secondary antibodies for 1 hour at room temperature. Protein bands were detected using enhanced chemiluminescence (ECL) reagents (Bio-Rad, USA) and imaged with a digital chemiluminescence system. All immunoblots were performed in at least three independent biological replicates.

### Immunofluorescence

For immunofluorescence staining, HUH-7 and Hep-3B cells were seeded at a density of 5 × 10^4^ cells per well onto cells were seeded onto coverslips placed in 24-well plates (1.9 cm² surface area per well) and allowed to adhere overnight. Cells were then fixed with 4% paraformaldehyde for 10 minutes at room temperature, followed by washing with PBS. Permeabilization was performed using 0.1% Triton X-100 in PBS for 20 minutes. After additional washes, cells were blocked with 5% donkey serum in PBS for 1 hour at room temperature to prevent nonspecific antibody binding. Primary antibodies against PKM1 (1:200;CST, 7067), GLUL (1:200; abcam, ab64613), HNF1A (1:100;CST, 89670)and HNF3A (1:100; CST, 58613) at 4°C were applied and incubated overnight at 4°C. The following day, cells were washed and incubated with Alexa Fluor-conjugated secondary antibodies for 1 hour at room temperature in the dark:Alexa Fluor 488-conjugated goat anti-rabbit IgG (CST, 4412) for PKM1 and HNF1A; Alexa Fluor 594-conjugated goat anti-mouse IgG (Abcam, ab150116) for GLUL; and Alexa Fluor 594-conjugated goat anti-rabbit IgG (CST, 8889) for HNF3A. Nuclei were counterstained with DAPI for 5 minutes. After final washes, coverslips were mounted using antifade mounting medium. All staining procedures were performed in triplicate, and experiments were repeated independently at least three times (n = 3). Fluorescent images were acquired using epifluorescence microscope under identical exposure settings across conditions.

### Xenograft mouse model

Animal experiments were conducted in accordance with institutional ethical guidelines. Male BALB/c nude mice (4–6 weeks old) were housed under specific pathogen-free (SPF) conditions with ad libitum access to food and water. For xenograft studies, HUH-7 cells (5 × 10^6^ cells/mouse) were subcutaneously injected into the right flank of each mouse. Based on our previous studies (31), the dosage regimen of MS275 (20 mg/kg, intraperitoneally every three days) was adopted. Mice were then randomly assigned to two groups: one group received MS275, while the control group received vehicle (DMSO or PBS) on the same schedule. Treatments continued for three weeks. Body weight and tumor size were monitored every 3 days, and tumor volume was calculated using the formula V = (length × width²)/2. At the experimental endpoint, mice were euthanized, and tumors were excised, weighed, and processed for downstream analyses including immunohistochemistry and western blotting. All animal experiments were conducted in accordance with the Animal Care and Use Principles approved by the Ethics Committee at Sun Yat-sen University Cancer Center (Approval ID: 2022001429).

### Immunohistochemistry

5 µm thick paraffin-embedded sections were deparaffinized, rehydrated through graded alcohols, and subjected to antigen retrieval using citrate buffer (pH 6.0) at high temperature. After blocking endogenous peroxidase activity with 3% hydrogen peroxide for 15 minutes, the sections were incubated with primary antibodies against Ki-67 (1:200; abcam, ab16667), PKM1 (1:100; CST, 7067), GLUL (1:100; abcam, ab64613), HNF1A (1:100; CST, 89670), and HNF3A (1:100; CST, 58613) overnight at 4°C. The following day, sections were incubated with horseradish peroxidase (HRP)-conjugated secondary antibodies and visualized using diaminobenzidine (DAB) substrate solution (Yeasen, China). Counterstaining was performed with hematoxylin. Slides were dehydrated, cleared, and mounted using standard procedures. Immunostaining was performed on tumor sections from at least three biological replicates per experimental group. Images were captured using a bright-field microscope under consistent settings.

### Statistical analysis

All experiments were independently repeated three times, and data were expressed as mean ± standard deviation (SD). Western blot (WB) band intensities were quantified using ImageJ from three independent blots. Statistical analyses were performed with GraphPad Prism 9.0 (GraphPad Software, USA). Comparisons between two groups were conducted using a two-tailed Student’s t-test, and multiple group comparisons were analyzed by two-way ANOVA followed by Bonferroni *post hoc* test. A p-value < 0.05 was considered statistically significant.

## Result

### HDAC inhibitors suppresses HCC cell growth by inducing cell cycle arrest and promoting differentiation

**Figure 1:**
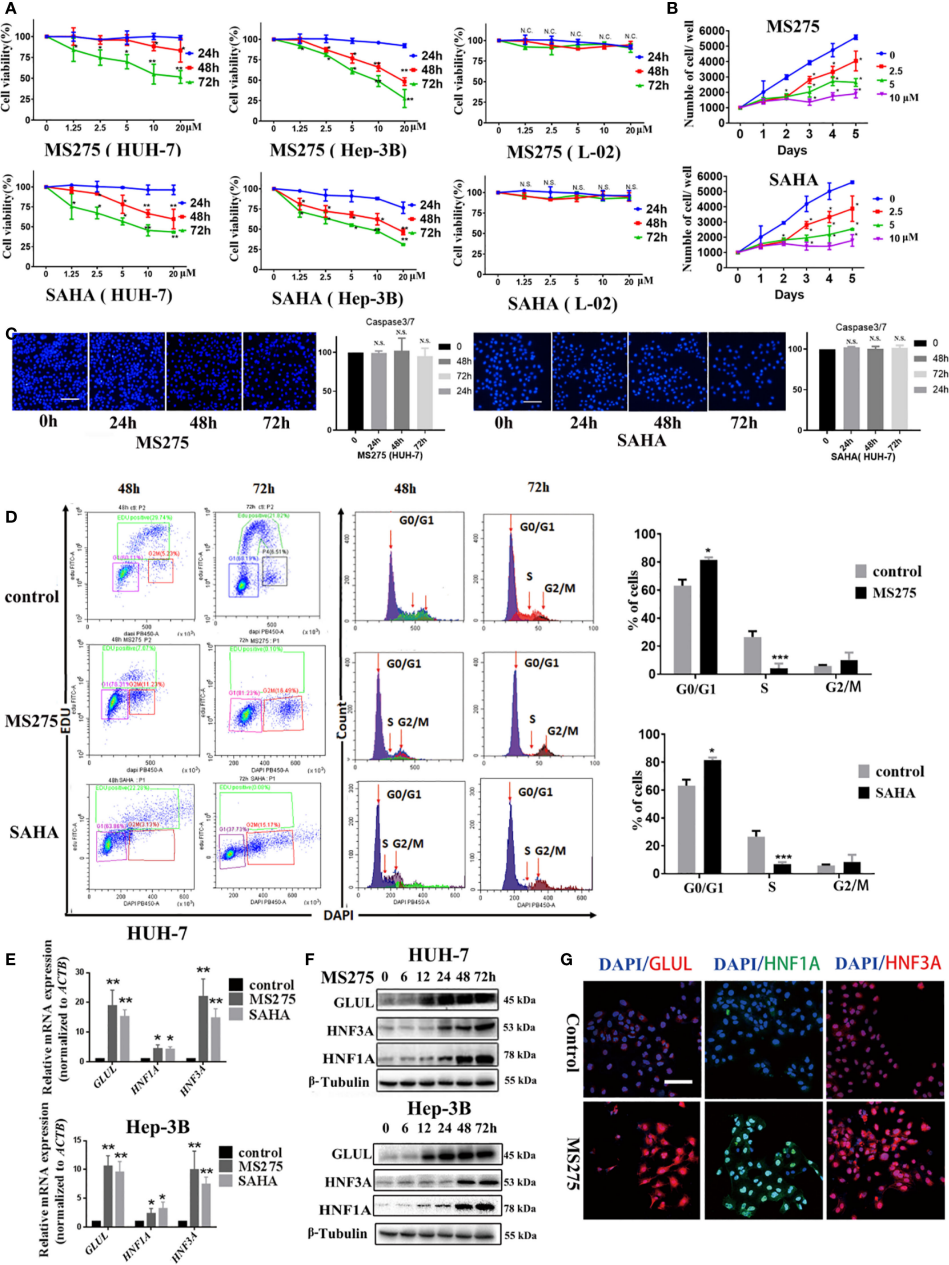
HDACi induces a G0/G1 phase cell cycle arrest and cell differentiation in HCC cells. **(A)** Cell viability analyses of HUH-7 and Hep-3B HCC cells treated with MS275 and SAHA at different concentrations for 24, 48, and 72 hours. **(B)** Cell proliferation assay showing the number of viable cells over 5 days following treatment with MS275 or SAHA at concentrations of 2.5, 5, and 10 µM, compared to the 0 µM control. **(C)** Immunofluorescence images of Caspase-3/7 combined with Hoechst 33342 nuclear staining in HUH-7 cells treated with MS275 or SAHA at different time points (24h, 48h, 72h). Corresponding quantification indicate no significant apoptosis induction. **(D)** Flow cytometry analysis using EDU and DAPI staining. **(E)** qRT-PCR analysis of mRNA expression levels of *GluL*, *HNF1A*, and *HNF3A* in HUH-7 and Hep-3B cells treated with MS275 or SAHA. **(F, G)** Immunofluorescence staining **(F)** and Western blot analysis **(G)** of GluL, HNF1A, and HNF3A in HUH-7 cells treated with MS275. Scale: 100 μm.;N.S., Not Significant; *p < 0.05, **p < 0.01, ***p < 0.001.

HDAC inhibitors, MS275 and SAHA, significantly reduced cell viability, with a more pronounced effect at higher concentrations and longer exposure times (**Figure 1A**). Moreover, both MS275 and SAHA significantly suppressed the proliferation in a dose-dependent manner (**Figure 1B**). To investigate whether these HDAC inhibitors induces apoptosis, To investigate whether these HDAC inhibitors induce apoptosis, we examined Caspase-3/7 activation and nuclear morphology. It revealed no appreciable increase in Caspase-3/7 signal, nuclear condensation, or fragmentation at 24, 48, or 72 h (**Figure 1C**), suggesting that MS275 and SAHA primarily suppressed proliferation rather than inducing cell apoptosis. Further analysis using flow cytometry revealed that MS275 and SAHA treatment led to a marked reduction in the percentage of cells in the S-phase, while an accumulation of cells in the G0/G1 phase was observed (**Figure 1D**), indicating that MS275 and SAHA inhibited cell cycle progression and thus suppressed proliferation. In addition to its role in inhibiting proliferation, MS275 was found to promote the differentiation of HCC cells. qRT-PCR analysis showed a significant increase in the mRNA levels of *GLUL*, *HNF1A*, and *HNF3A* following MS275 and SAHA treatment in both HUH-7 and Hep-3B cells (**Figure 1E**). Immunofluorescence staining and western blot analysis further confirmed the upregulation of these differentiation markers (**Figures 1F, G**), suggesting that MS275 not only suppressed HCC cell proliferation but also induces a more differentiated hepatocyte-like phenotype. Taken gather, these findings indicated that HDAC inhibitors inhibited the growth of HCC cells primarily by reducing the population of proliferating cells and inducing cell cycle arrest in the G0/G1 phase, with a significant decrease in the S-phase population. Additionally, HDAC inhibitors promoted cell differentiation by upregulating hepatocyte-specific markers, suggesting that HDAC inhibition may play a dual role in suppressing tumor growth and inducing hepatocyte differentiation, which could have therapeutic implications for hepatocellular carcinoma treatment.

### MS275 reprogram metabolic pathways and enhance energy metabolism in HCC cells

**Figure 2:**
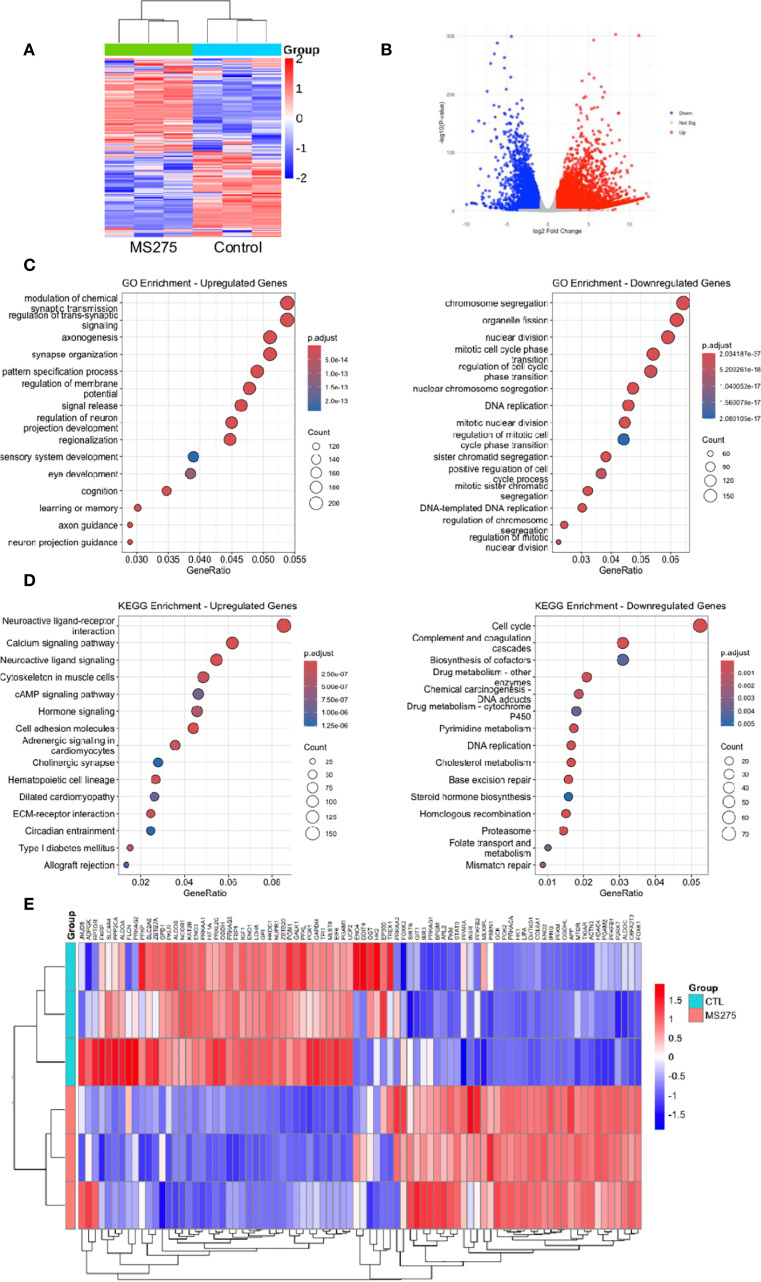
Transcriptome profiles reveal enhanced energy metabolism in MS275-treated cells. **(A, B)** Heatmap **(A)** and Volcano plot **(B)** illustrating DEGs in MS275-treated HCC cells, with significantly upregulated (red) and downregulated (green) genes compared to controls. **(C)** GO enrichment analysis of upregulated and downregylated DEGs. **(D)** KEGG pathway analysis of upregulated and downregylated DEGs. **(E)** A heatmap of differentially expressed genes related to metabolic processes.

Although both MS275 and SAHA inhibited proliferation and promoted differentiation in HCC cells, we focused primarily on MS275 due to its selective inhibition of Class I HDACs, which offers a more targeted epigenetic approach compared to the broader activity of SAHA, a pan-HDAC inhibitor that inhibits both Class I and Class II HDACs (32, 33). To gain deeper insight into the mechanisms underlying MS275-mediated effects in HCC, we performed RNA-seq analysis on MS275-treated cells. Transcriptome analysis revealed that MS275 induce significant changes in gene expression. A total of 7791 DEGs were identified, with 4843upregulated genes and 2948 downregulated genes (**Figures 2A, B**). GO enrichment analysis of differentially expressed genes in MS275-treated HCC cells revealed a clear functional divergence between upregulated and downregulated gene sets (**Figure 2C**). Upregulated genes were significantly enriched in biological processes related to neuronal function and intercellular signaling, including modulation of chemical synaptic transmission, regulation of trans-synaptic signaling, axonogenesis, synapse organization, and regulation of membrane potential. These results suggest that MS275 may induce transcriptional programs associated with enhanced cellular communication or neuronal-like differentiation. In contrast, downregulated genes were predominantly involved in mitotic processes and genomic maintenance, such as chromosome segregation, organelle fission, nuclear division, DNA replication, and mitotic nuclear division. This pattern indicates a suppression of cell cycle progression and proliferative activity in response to MS275. Collectively, these findings highlight that MS275 treatment shifts the cellular transcriptional landscape away from proliferation and toward specialized signaling and potentially differentiated states. KEGG pathway enrichment analysis revealed significant functional divergence between upregulated and downregulated genes in MS275-treated HCC cells (**Figure 2D**). Upregulated genes were predominantly enriched in pathways related to cellular signaling and communication. Interestingly, pathways included cytoskeleton regulation in muscle cells, cell adhesion molecules, and adrenergic signaling in cardiomyocytes, all pointing toward enhanced intercellular signaling, structural reorganization, and potential shifts in cell identity. Conversely, downregulated genes were significantly associated with pathways essential for cell proliferation, DNA maintenance, and metabolic homeostasis. These included cell cycle, DNA replication, complement and coagulation cascades, base excision repair, homologous recombination, and mismatch repair. Additionally, pathways such as cholesterol metabolism, steroid hormone biosynthesis, and biosynthesis of cofactors were also suppressed. Together, these findings suggest that MS275 treatment suppresses proliferative and metabolic gene programs while enhancing signaling and structural pathways, consistent with a shift toward a more differentiated or regulated cellular state. Both GO and KEGG analyses revealed a dyresgulated metabolic processes after MS275 treatment. The heatmap revealed a pronounced alteration in the expression of genes associated with glycolysis, indicating significant transcriptional reprogramming of glycolytic pathways in response to treatment (**Figure 2E**). These findings provide further evidence for a shift in energy metabolism, which may underlie the observed phenotypic responses in the cells subjected to treatment.

Considering that the GO and KEGG pathway analyses consistently showed strong enrichment in metabolic processes and hepatocyte differentiation, we further explored the functional implications of these gene expression changes (**Supplementary Figure S1**). Specifically, metabolic GO terms such as glucose catabolic process, fatty acid oxidation, and lipid biosynthesis were significantly enriched, indicating a broad reprogramming of cellular metabolism in response to MS275. In parallel, enrichment in chromatin-related processes, including histone modification, chromatin remodeling, and protein-containing complex remodeling, suggests that epigenetic regulation may be a central mechanism driving these transcriptional changes. Importantly, the DEGs involved in liver development, regeneration, and hepatocyte differentiation implies that MS275 may promote hepatic lineage commitment or restore hepatic identity. Together, these findings support the hypothesis that MS275 treatment modulates both the metabolic and epigenetic landscape to drive functional re-differentiation in HCC cells.

### MS275 reprograms metabolism in HCC cells by enhancing both glycolysis and oxidative phosphorylation

**Figure 3:**
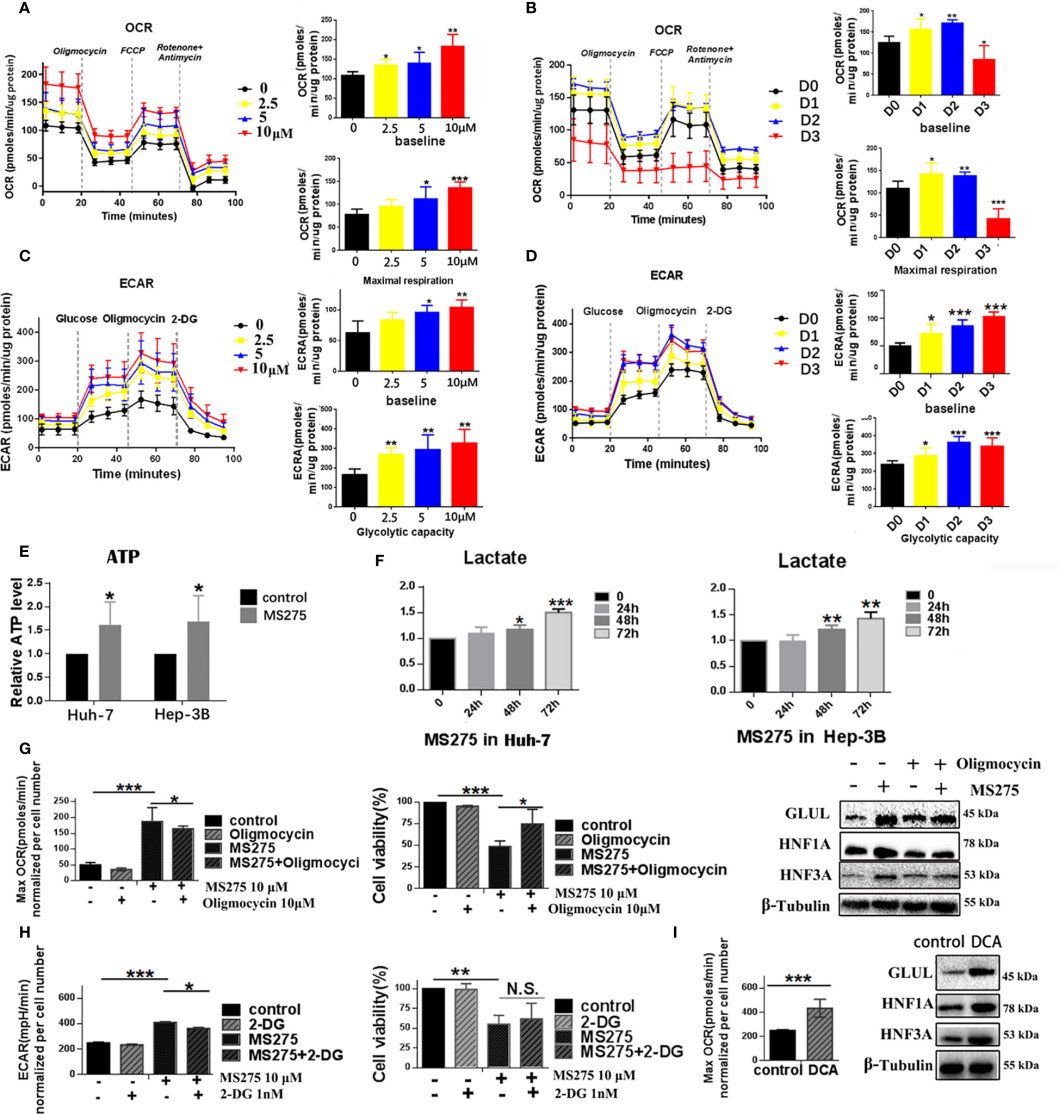
HDACi MS275 reprogramme the glycometabolism, characterized by stimulated glycolysis and oxidative phosphorylation. **(A, B)** OCR measurements in HUH-7 **(A)** and Hep-3B **(B)** cells treated with different concentrations of MS275, demonstrating increased baseline and maximal respiration in a dose- and time-dependent manner. **(C, D)** ECAR measurements in HUH-7 **(C)** and Hep-3B **(D)** cells treated with MS275, showing an increase in glycolysis and glycolytic capacity in a dose- and time-dependent manner. **(E)** Relative ATP levels in HUH-7 and Hep-3B cells, showing increased ATP production in response to MS275 treatment. **(F)** Lactate production in HUH-7 and Hep-3B cells, demonstrating an induction in glycolysis-derived lactate following MS275 treatment over time. **(G)** Effect of the oxidative phosphorylation inhibitor oligomycin on MS275-treated cells, showing reduced OCR and cell viability. **(H)** Effect of the glycolysis inhibitor 2-DG on MS275-treated cells, indicating that glycolysis inhibition further reduces ECAR but did not significantly affect MS275-induced growth suppression at low doses. **(I)** Treatment with the mitochondrial pyruvate carrier inhibitor DCA increased maximal OCR and the expression of metabolic markers (GLUL, HNF3A, and HNF1A) *: p<0.05 ; **: p<0.01; ***: p<0.001.

To investigate the metabolic effects of MS275, we assessed mitochondrial respiration and glycolysis in HCC cell lines Huh-7 and Hep-3B using Seahorse extracellular flux analysis. Treatment with increasing concentrations of MS275 (0 µM, 2.5 µM, 5 µM, and 10 µM) led to a progressive elevation in mitochondrial respiration in Huh-7 cells. The OCR time course showed significant increases in both baseline and maximal respiration, with the highest effect observed at 10 µM (**Figure 3A**). Quantification confirmed statistically significant enhancement in OCR at all doses, especially at 5 µM and 10 µM. These results suggest that MS275 promotes oxidative phosphorylation in a dose-dependent manner. Interestingly, when the OCR was assessed at Day 1, Day 2, and Day 3 post-treatment with MS275, it showed a different pattern (**Figure 3B**). While Day 1 and Day 2 showed increased OCR, consistent with initial mitochondrial activation, Day 3 revealed a marked decline in both baseline and maximal respiration. This suggests that MS275 initially boosts mitochondrial activity, but prolonged exposure may lead to mitochondrial exhaustion or a shift away from oxidative metabolism. In parallel, ECAR measurements revealed a dose-dependent enhancement in glycolytic activity upon MS275 treatment. Glycolytic capacity was significantly increased at 5 µM and 10 µM, indicating that MS275 reprograms cells toward greater reliance on glycolysis, potentially as a metabolic adaptation to HDAC inhibition. This is consistent with an energy-demanding process such as cellular differentiation (**Figure 3C**). Unlike the OCR trend, ECAR remained elevated throughout MS275 treatment from Day 1 to Day 3 (**Figure 3D**). Both baseline glycolysis and glycolytic capacity showed a steady increase over time, with the most significant elevation observed on Day 3. This suggests that even as mitochondrial respiration declines with prolonged treatment, cells compensate by upregulating glycolysis, indicating a metabolic shift toward glycolytic dependence. The inability of 2-DG (the glycolysis inhibitor) to fully reverse this effect suggests additional mechanisms or compensatory pathways are involved in the metabolic rewiring induced by HDAC inhibition. To further confirm these metabolic changes, ATP production and lactate levels were assessed. ATP levels increased upon MS275 treatment, consistent with enhanced oxidative phosphorylation (**Figure 3E**). Additionally, lactate production was significantly reduced in a time-dependent manner, further supporting the induction of glycolysis (**Figure 3F**). Oligomycin treatment reduced OCR and significantly increased cell viability in MS275-treated cells (**Figure 3G**), indicating that MS275 enhances mitochondrial respiration. Western blot analysis showed that Oligomycin decreased the MS275-induced differentiation markers, including GLUL, HNF3A, and HNF1A. Similarly, treatment with the glycolysis inhibitor 2-DG further reduced ECAR but had minimal impact on MS275-induced growth suppression at low doses, suggesting that glycolysis inhibition alone was not the primary mechanism of MS275’s effects (**Figure 3H**). Mitochondrial metabolism was further assessed using DCA, an inhibitor of mitochondrial pyruvate import (34). DCA treatment significantly increased maximal OCR and the expression of key metabolic differentiation markers, including GLUL, HNF3A, and HNF1A (**Figure 3I**), indicating that mitochondrial metabolism plays a crucial role in MS275-induced hepatocyte differentiation. These findings suggest that the increase in oxidative metabolism, rather than glycolysis, is responsible for MS275-induced differentiation and proliferation inhibition in HCC cells. Together, these findings suggest that MS275 induces a metabolic state favoring oxidative metabolism, which is essential for its differentiation-promoting and anti-proliferative effects in HCC cells.

### MS275 induces ROS production via enhanced oxidative phosphorylation, leading to differentiation and loss of proliferation in HCC cells

**Figure 4:**
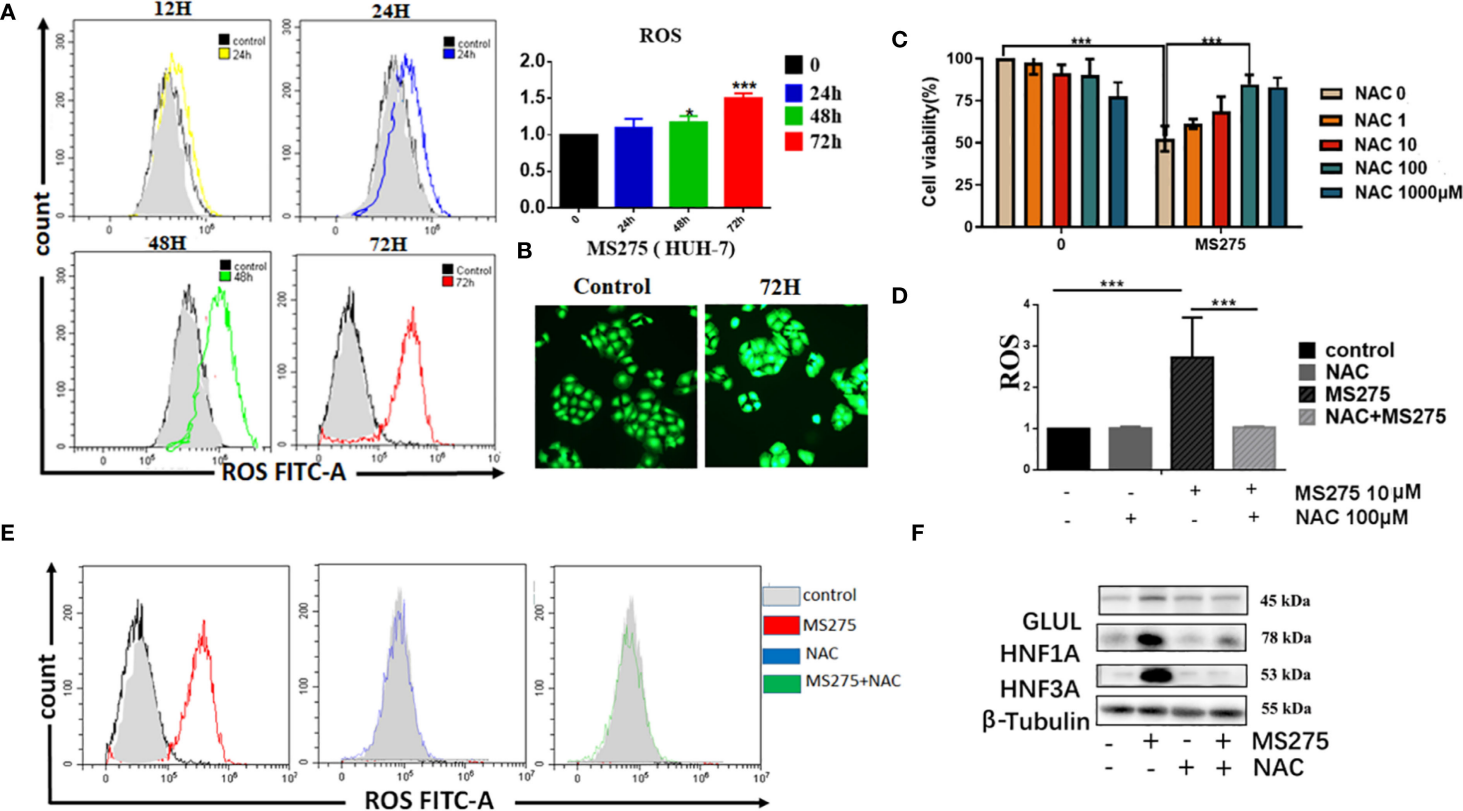
ROS production by boosted oxygen phosphorylation is responsible for MS275 induced differentiation and proliferation-loss. **(A)** Flow cytometry analysis of ROS levels in HUH-7 cells treated with MS275 for 12, 24, 48, and 72 hours, showing a time-dependent increase in ROS production. **(B)** Immunofluorescence images of ROS in control and MS275-treated cells at 72 hours, showing elevated ROS levels. **(C)** Cell viability assay demonstrating that MS275 reduced cell viability, while NAC partially rescued cell survival, suggesting that ROS contributes to MS275-induced proliferation inhibition. **(D)** Quantification of ROS levels in cells treated with MS275 alone or co-treated with NAC, showing that NAC effectively reduced ROS production. **(E)** Flow cytometry analysis of mitochondrial ROS levels in control, MS275, NAC, and MS275+NAC-treated cells, confirming that MS275-induced ROS was mitochondrial-derived. **(F)** Western blot analysis of differentiation markers (GLUL, HNF1A, HNF3A) in control and MS275-treated cells, with or without NAC, showing that ROS inhibition suppressed MS275-induced differentiation *: p<0.05; ***: p<0.001.

Flow cytometry analysis showed that MS275 treatment resulted in a significant, time-dependent increase in ROS levels in HCC cells (**Figure 4A**). Quantification of ROS confirmed that levels progressively increased at 24, 48, and 72 hours post-treatment. Fluorescence imaging further validated this increase, with a strong ROS signal observed in MS275-treated cells at 72 hours (**Figure 4B**). To determine whether ROS plays a functional role in MS275-induced effects, cells were co-treated with NAC, a ROS scavenger (35). NAC partially rescued cell viability in MS275-treated cells (**Figure 4C**), suggesting that ROS production contributed to MS275-induced growth suppression. Moreover, NAC treatment effectively suppressed MS275-induced ROS accumulation (**Figure 4D**), indicating that MS275 enhanced oxidative stress through mitochondrial respiration. Further analysis demonstrated that MS275 significantly increased mitochondrial ROS levels, while NAC treatment effectively reduced ROS back to baseline levels (**Figure 4E**). Western blot analysis revealed that MS275 upregulated GLUL, HNF1A, and HNF3A (**Figure 4F**). However, co-treatment with NAC diminished this upregulation, indicating that ROS production was required for MS275-induced differentiation. Together, these findings demonstrate that MS275 enhances mitochondrial oxidative phosphorylation, leading to increased ROS production, which in turn drives HCC cell differentiation and inhibits proliferation.

### MS275 induces PKM1 expression to enhance oxidative phosphorylation, ROS production, and cell differentiation in HCC cells

**Figure 5:**
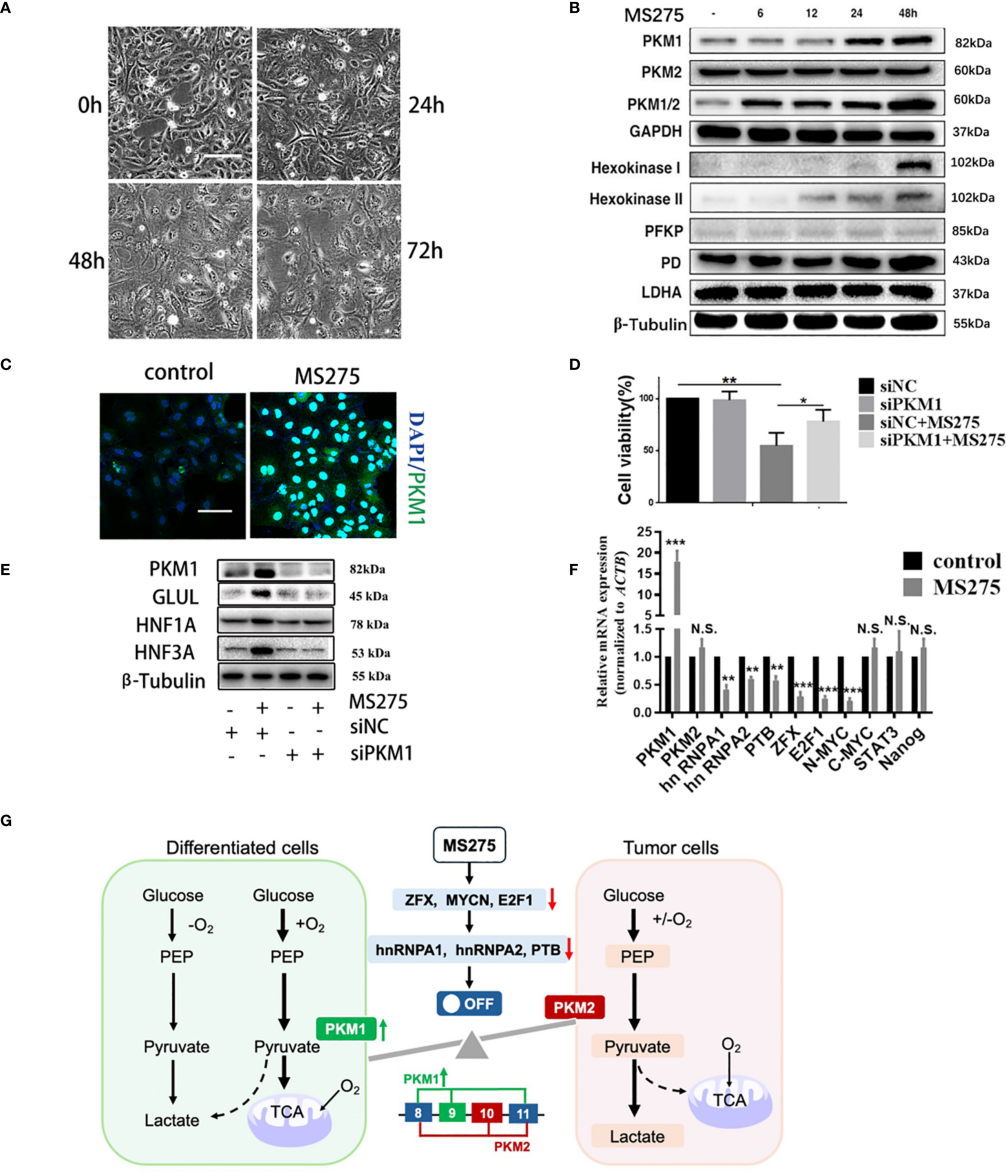
PKM1 expression contributes to ROS production and cell differentiation by MS275. **(A)** Morphological changes over time (0h, 24h, 48h, 72h) after MS275 treatment. **(B)** Western blot analysis showing time-dependent upregulation of PKM1 and metabolic enzymes involved in oxidative phosphorylation and glycolysis in MS275-treated cells. **(C)** Immunofluorescence images showing increased PKM1 expression in MS275-treated cells compared to controls. **(D)** Cell viability assay showing reduced proliferation upon MS275 treatment, suggesting that PKM1 upregulation contributed to growth inhibition. **(E)** Western blot analysis of PKM1, GLUL, HNF1A, and HNF3A in control, MS275-treated, siPKM1, and siPKM1+MS275-treated cells, indicating that *PKM1* knockdown blocked MS275-induced differentiation. **(F)** qRT-PCR analysis of metabolic and differentiation-related genes in control and MS275-treated cells, showing significant upregulation of *PKM1* and differentiation markers. **(G)** Schematic representation of PKM1’s role in oxidative phosphorylation and glycolysis, showing how PKM1-mediated oxidative metabolism promotes differentiation and inhibits proliferation in HCC cells *: p<0.05 ; **: p<0.01; ***: p<0.001. "N.S" means not significant.

We observed that MS275 treatment led to significant morphological changes in HCC cells over time (**Figure 5A**). Western blot analysis revealed that MS275 treatment upregulated PKM1, the key isoform of pyruvate kinase associated with oxidative metabolism (36), while PKM2 expression remained unchanged (**Figure 5B**). Additionally, enzymes involved in glycolysis and oxidative phosphorylation, including hexokinase I, hexokinase II, PFKP, and PD, were elevated, while LDHA did not change. Immunofluorescence staining indicated an increase in PKM1 expression following MS275 treatment (**Figure 5C**). To determine whether PKM1 plays a functional role in MS275-induced effects, *PKM1* was knocked down using siRNA (siPKM1). CCK-8 assays demonstrated that MS275 significantly reduced cell viability, while knockout of *PKM1* restraint this reduction (**Figure 5D**), indicating that MS275 suppressed cell viability by up-regulating PKM1. Western blot analysis demonstrated that *PKM1* knockdown prevented MS275-induced upregulation of hepatocyte differentiation markers GLUL, HNF1A, and HNF3A (**Figure 5E**), indicating that PKM1 is required for differentiation. qRT-PCR analysis showed that MS275 upregulated *PKM1* and metabolic genes involved in oxidative phosphorylation, while genes related to glycolysis remained largely unchanged (**Figure 5F**). Taken together, these results illustrate that PKM1-mediated oxidative phosphorylation is associated to cell differentiation and metabolic reprogramming (**Figure 5G**). MS275 promotes HCC cell differentiation and inhibits proliferation by upregulating PKM1, thereby enhancing oxidative phosphorylation and ROS production.

### MS275 suppresses tumor growth and promotes hepatocyte differentiation in HCC xenografts

**Figure 6:**
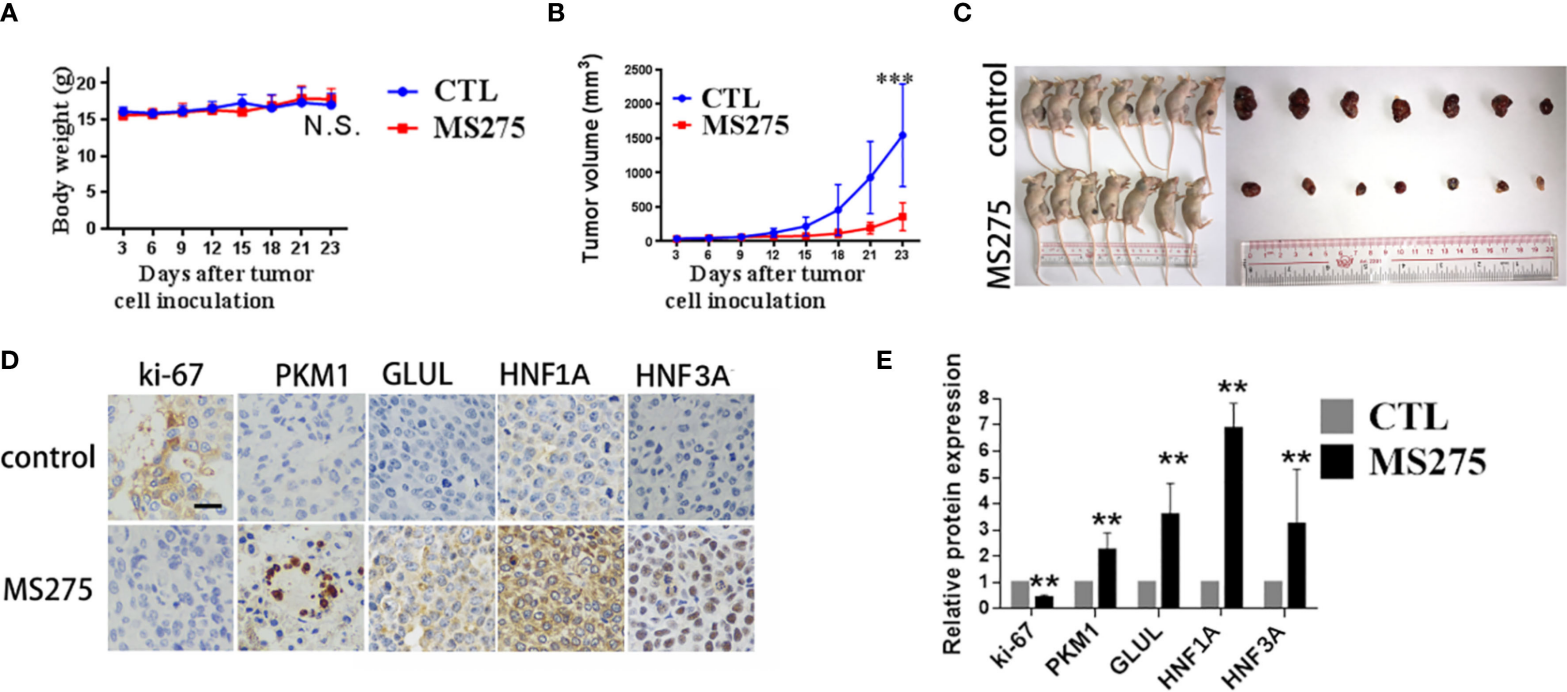
MS275 treatment represses tumor growth and induces differentiation in HCC xenograft models. **(A)** Body weight measurements of mice showing no significant difference between control (CTL) and MS275-treated groups. **(B)** Tumor volume measurements over time, demonstrating a significant reduction in tumor growth in MS275-treated mice compared to controls. **(C)** Representative images of xenograft-bearing mice and excised tumors, showing markedly smaller tumors in the MS275-treated group. **(D)** Immunohistochemical staining of tumor tissues for Ki-67, PKM1, GLUL, HNF1A, and HNF3A, showing reduced Ki-67 expression and increased differentiation markers in the MS275-treated group. **(E)** Quantification of protein expression levels from IHC staining, indicating significant upregulation of differentiation markers (PKM1, GLUL, HNF1A, HNF3A) and downregulation of the proliferation marker Ki-67 in MS275-treated tumors. Scale:100 μm.;N.S., Not Significant; **p < 0.01, ***p < 0.001.

To evaluate the effects of MS275 on tumor growth *in vivo*, we established a HCC xenograft model. Mice treated with MS275 showed no significant change in body weight compared to controls (**Figure 6A**), suggesting that MS275 did not cause systemic toxicity. However, tumor volume measurements revealed that MS275 treatment significantly reduced tumor size compared to controls (**Figures 6B, C**), indicating MS275 suppressed tumor growth. Ki-67 staining demonstrated a significant reduction in proliferating cells in MS275-treated tumors (**Figures 6D, E**), suggesting that MS275 inhibits tumor cell proliferation. Additionally, PKM1 expression was significantly increased, along with the hepatocyte differentiation markers GLUL, HNF1A, and HNF3A (**Figures 6D, E**), indicating that MS275 promoted differentiation in HCC cells. These results demonstrate that MS275 effectively suppresses tumor growth in HCC xenografts by inhibiting proliferation and promoting differentiation, without causing systemic toxicity.

## Discussion

In this study, we identified the Class I HDAC inhibitor MS275 as a potent suppressor of HCC cell proliferation and a promoter of hepatocyte-like differentiation. Our findings reveal that MS275 treatment results in cell cycle arrest at the G0/G1 phase. Importantly, MS275 induces the expression of key hepatic markers such as HNF1A, HNF3A, and GLUL, supporting a shift toward a more differentiated phenotype. Transcriptomic analysis further corroborates these effects, showing activation of hepatocyte-specific gene signatures and metabolic remodeling. These results reveal MS275’s dual capacity to inhibit tumor growth and promote cellular differentiation, suggesting its promise as a differentiation-based therapeutic strategy for HCC.

In HCC, MS275 has demonstrated antitumor effects through inducing apoptosis and potentially enhance the immune response against cancer cells (19, 20). Here, we also observed MS275 significantly inhibited proliferation and induced cell cycle arrest in the G0/G1 phase. Studies show that MS275 induces cell cycle arrest in the G0/G1 phase, a stage where cells are typically quiescent or preparing for DNA replication. Previous study has shown that MS275 induces cell cycle arrest and apoptosis on malignant ascites cells and prostate cancer cells (37, 38). Interestingly, MS275 induced cell cycle arrest without triggering apoptosis in HCC cells. Importantly, MS275 also triggered a differentiation program in HCC cells. This was evidenced by increased expression of hepatocyte-specific markers including GLUL, HNF1A, and HNF3A, at both the mRNA and protein levels. Moreover, MS275 treated cells exhibited morphological changes indicative of maturation, consistent with a differentiated HCC phenotype, which is closely resemble normal hepatocytes, exhibiting minimal nuclear atypia and intact cell membranes (39). Cell cycle analysis revealed significant accumulation of cells in the G1 phase, supporting the concept that MS275 promotes cell cycle exit, which is a key feature of terminal differentiation (40). HCC differentiation is commonly associated with a permanent exit from the proliferative cycle and entry into a quiescent, functionally mature state (41).These phenotypic and molecular changes collectively indicate that MS275 promotes a shift from a malignant to a more differentiated cellular state. Well-differentiated tumors tend to grow more slowly and respond better to therapy, while poorly differentiated tumors are more aggressive and resistant (1, 39). Therefore, differentiation therapy, which aims to reprogram malignant cells toward a more normal, differentiated phenotype, is an area of active research with substantial therapeutic potential (42). Our results suggest that MS275 could induces a more mature, hepatocyte-like phenotype, a process that could help attenuate tumor aggressiveness and improve therapeutic response.

Transcriptomic and functional metabolic analysis revealed that MS275 induces a metabolic shift from glycolysis to oxidative phosphorylation. This shift was associated with increased ATP production, decreased lactate levels, and a reduction in glycolytic flux (lower ECAR). At the core of this reprogramming is PKM1, a pyruvate kinase isoform known for promoting oxidative metabolism and differentiation (36). MS275 significantly upregulated PKM1 expression without affecting PKM2. Knockdown of PKM1 using siRNA abolished MS275-induced growth suppression and differentiation, proving that PKM1 is essential for these effects. This reveals a novel PKM1-mediated association between metabolism and differentiation in HCC. In glioblastoma, we have demonstrated that activation of the cAMP-PGC1α pathway reverses the Warburg effect, promoting mitochondrial biogenesis and a shift toward oxidative phosphorylation, which drives differentiation into astrocytes (43). Similarly, in the this study, MS275 treatment enhances oxidative phosphorylation and induces a more differentiated phenotype in HCC cells, suggesting that metabolic remodeling may serve as a key mechanism linking epigenetic modulation to differentiation.

ROS have a dual role in regulating cell fate, acting as key signaling molecules that can drive differentiation at controlled levels, but inducing cytotoxicity and impairing differentiation when elevated excessively (44, 45). The shift to mitochondrial metabolism was accompanied by a dose- and time-dependent increase in ROS. These ROS, generated through enhanced OXPHOS, played a crucial role in mediating differentiation: co-treatment with the ROS scavenger NAC reduced both ROS levels and differentiation marker expression, and partially rescued cell viability. Thus, ROS function as signaling molecules that translate metabolic changes into gene expression programs favoring differentiation. Our previous has showed that the Syk inhibitor R406 enhances oxidative phosphorylation and elevates ROS, leading to anti-tumor effects in glioma stem cells. This supports the role of ROS in inducing cell cycle arrest and promoting differentiation (46). However, the downstream pathways through which ROS exert these effects remain to be clarified.

*In vivo*, MS275 effectively suppressed tumor growth in a HCC xenograft model without causing systemic toxicity, as evidenced by stable body weight. Tumors showed reduced proliferation and increased expression of PKM1 and hepatocyte differentiation markers, confirming the dual action of MS275 in promoting differentiation and limiting proliferation. These findings position MS275 as a multi-modal therapeutic agent that not only halts growth but also reprograms metabolism and promotes differentiation. This is particularly relevant in HCC, where drug resistance and tumor heterogeneity limit the success of standard therapies (47).

Our findings position MS275 as a promising epigenetic therapy for HCC, acting through both metabolic and differentiation pathways. While HDAC inhibitors are known to induce apoptosis and cell cycle arrest in various cancers (37, 38), this study reveals novel insight by elucidating a PKM1-ROS-dependent mechanism of differentiation. Given that differentiation therapy has had clinical success in other cancers (e.g., ATRA in acute promyelocytic leukemia), similar strategies may be effective in liver cancer where poor differentiation correlates with worse prognosis (48). Sorafenib is an oral multikinase inhibitor that has been widely used as a first-line systemic therapy for advanced HCC, particularly in cases that are unresectable. It was the first drug to demonstrate a statistically significant improvement in overall survival in patients with advanced HCC (49). Mechanistically, sorafenib exerts its antitumor effects by targeting multiple kinases, including Raf kinases, VEGFR, and PDGFR, thereby inhibiting tumor cell proliferation and angiogenesis (50). However, sorafenib primarily provides cytostatic effects rather than inducing tumor cell differentiation. In our study, the focus was on MS275’s ability to promote differentiation and metabolic reprogramming in HCC, a mechanism that is fundamentally distinct from sorafenib’s. Importantly, resistance to sorafenib remains a clinical challenge, and its modest survival benefit underscores the need for alternative or complementary therapies (51). Given the non-overlapping mechanisms of sorafenib and MS275, one targeting oncogenic signaling pathways and angiogenesis, the other modulating gene expression through epigenetic regulation, future studies should investigate the potential for combination therapy. This dual approach may enhance treatment efficacy by simultaneously suppressing tumor growth and promoting differentiation, possibly overcoming some limitations of current monotherapies.

Despite the promising data, several limitations should be addressed. First, the mechanism by which ROS promote differentiation remains unclear. Identifying redox-sensitive transcription factors or signaling pathways that drive hepatic differentiation would deepen our understanding. Second, while MS275 showed efficacy in xenografts, clinical translation will require optimization of dosing, scheduling, and possibly combination therapies to enhance efficacy while minimizing side effects. Third, our study did not assess the immune-related effects of MS275. Given the emerging relevance of HDAC inhibition in reshaping anti-tumor immunity, future work should incorporate immune-competent models to evaluate the potential immunomodulatory impact of MS275 in hepatocellular carcinoma.

## Conclusion

MS275 suppresses HCC cell proliferation and promotes differentiation by enhancing oxidative phosphorylation and ROS production via PKM1 upregulation. These findings reveal its potential as a differentiation-based therapeutic strategy for hepatocellular carcinoma.

## Data Availability

The raw data supporting the conclusions of this article will be made available by the authors, without undue reservation.

## Supplementary material

Supplementary Figure 1 GO enrichment analysis highlights key biological processes altered by MS275 treatment. (A-D) GO terms related to metabolism **(A)**, cancer-related processes **(B)**, chromatin remodeling processes **(C)**, and hepatocyte differentiation-related processes **(D)**.

## References

[1] Asafo-Agyei KO Samant H. Hepatocellular Carcinoma. Treasure Island (FL: StatPearls (2025). 32644603

[2] Moon AM Singal AG Tapper EB. Contemporary epidemiology of chronic liver disease and cirrhosis. Clin Gastroenterol Hepatol. (2020) 18:2650–66. doi: 10.1016/j.cgh.2019.07.060, PMID: 31401364 PMC7007353

[3] Abdelhamed W El-Kassas M. Hepatocellular carcinoma recurrence: Predictors and management. Liver Res. (2023) 7:321–32. doi: 10.1016/j.livres.2023.11.004, PMID: 39958776 PMC11791921

[4] Filgueira NA. Hepatocellular carcinoma recurrence after liver transplantation: Risk factors, screening and clinical presentation. World J Hepatol. (2019) 11:261–72. doi: 10.4254/wjh.v11.i3.261, PMID: 30967904 PMC6447422

[5] Wang H Chen X Calvisi DF. Hepatocellular carcinoma (HCC): the most promising therapeutic targets in the preclinical arena based on tumor biology characteristics. Expert Opin Ther Targets. (2021) 25:645–58. doi: 10.1080/14728222.2021.1976142, PMID: 34477018 PMC8511244

[6] Wang G Jiang X Torabian P Yang Z. Investigating autophagy and intricate cellular mechanisms in hepatocellular carcinoma: Emphasis on cell death mechanism crosstalk. Cancer Lett. (2024) 588:216744. doi: 10.1016/j.canlet.2024.216744, PMID: 38431037

[7] Fabregat I. Dysregulation of apoptosis in hepatocellular carcinoma cells. World J Gastroenterol. (2009) 15:513–20. doi: 10.3748/wjg.15.513, PMID: 19195051 PMC2653340

[8] Wu X Cao J Wan X Du S. Programmed cell death in hepatocellular carcinoma: mechanisms and therapeutic prospects. Cell Death Discov. (2024) 10:356. doi: 10.1038/s41420-024-02116-x, PMID: 39117626 PMC11310460

[9] Gallinari P Di Marco S Jones P Pallaoro M Steinkühler C. HDACs, histone deacetylation and gene transcription: from molecular biology to cancer therapeutics. Cell Res. (2007) 17:195–211. doi: 10.1038/sj.cr.7310149, PMID: 17325692

[10] Freese K Seitz T Dietrich P Lee SML Thasler WE Bosserhoff A. Histone deacetylase expressions in hepatocellular carcinoma and functional effects of histone deacetylase inhibitors on liver cancer cells *in vitro* . Cancers (Basel). (2019) 11:1587. doi: 10.3390/cancers11101587, PMID: 31635225 PMC6826839

[11] Zhang J Zhong Q. Histone deacetylase inhibitors and cell death. Cell Mol Life Sci. (2014) 71:3885–901. doi: 10.1007/s00018-014-1656-6, PMID: 24898083 PMC4414051

[12] Li Y Seto E. HDACs and HDAC inhibitors in cancer development and therapy. Cold Spring Harb Perspect Med. (2016) 6:a026831. doi: 10.1101/cshperspect.a026831, PMID: 27599530 PMC5046688

[13] Yamashita Y Shimada M Harimoto N Rikimaru T Shirabe K Tanaka S. Histone deacetylase inhibitor trichostatin A induces cell-cycle arrest/apoptosis and hepatocyte differentiation in human hepatoma cells. Int J Cancer. (2003) 103:572–6. doi: 10.1002/ijc.10699, PMID: 12494463

[14] Yan S Chen L Zhuang H Yang H Yang Y Zhang N. HDAC inhibition sensitize hepatocellular carcinoma to lenvatinib via suppressing AKT activation. Int J Biol Sci. (2024) 20:3046–60. doi: 10.7150/ijbs.93375, PMID: 38904018 PMC11186361

[15] Connolly RM Rudek MA Piekarz R. Entinostat: a promising treatment option for patients with advanced breast cancer. Future Oncol. (2017) 13:1137–48. doi: 10.2217/fon-2016-0526, PMID: 28326839 PMC5618943

[16] Tang D Mao Z Chen S Su M Lan S Yan R. MS275 induces tumor immunosuppression by upregulating PD-L1 and enhances the efficacy of anti-PD-1 immunotherapy in colorectal cancer. Cancer Immunol Immunother. (2025) 74:150. doi: 10.1007/s00262-025-04004-4, PMID: 40095110 PMC11914531

[17] Stubbs MC Kim W Bariteau M Davis T Vempati S Minehart J. Selective inhibition of HDAC1 and HDAC2 as a potential therapeutic option for B-ALL. Clin Cancer Res. (2015) 21:2348–58. doi: 10.1158/1078-0432.CCR-14-1290, PMID: 25688158 PMC4433811

[18] Rosato RR Almenara JA Grant S. The histone deacetylase inhibitor MS-275 promotes differentiation or apoptosis in human leukemia cells through a process regulated by generation of reactive oxygen species and induction of p21CIP1/WAF1 1. Cancer Res. (2003) 63:3637–45., PMID: 12839953

[19] Xiao W Dong W Zhang C Saren G Geng P Zhao H. Effects of the epigenetic drug MS-275 on the release and function of exosome-related immune molecules in hepatocellular carcinoma cells. Eur J Med Res. (2013) 18:61. doi: 10.1186/2047-783X-18-61, PMID: 24359553 PMC3881022

[20] Gahr S Peter G Wissniowski TT Hahn EG Herold C Ocker M. The histone-deacetylase inhibitor MS-275 and the CDK-inhibitor CYC-202 promote anti-tumor effects in hepatoma cell lines. Oncol Rep. (2008) 20:1249–56., PMID: 18949429

[21] Ryan QC Headlee D Acharya M Sparreboom A Trepel JB Ye J. Phase I and pharmacokinetic study of MS-275, a histone deacetylase inhibitor, in patients with advanced and refractory solid tumors or lymphoma. J Clin Oncol. (2005) 23:3912–22. doi: 10.1200/JCO.2005.02.188, PMID: 15851766

[22] Bitzer M Horger M Giannini EG Ganten TM Wörns MA Siveke JT. Resminostat plus sorafenib as second-line therapy of advanced hepatocellular carcinoma - The SHELTER study. J Hepatol. (2016) 65:280–8. doi: 10.1016/j.jhep.2016.02.043, PMID: 26952006

[23] He Y Chen D Yi Y Zeng S Liu S Li P. Histone Deacetylase Inhibitor Sensitizes ERCC1-High Non-small-Cell Lung Cancer Cells to Cisplatin via Regulating miR-149. Mol Ther Oncolytics. (2020) 17:448–59. doi: 10.1016/j.omto.2020.05.001, PMID: 32478168 PMC7251316

[24] Chiu CF Guerrero JJG Regalado RRH Zhou J Notarte KI Lu YW. Insights into metabolic reprogramming in tumor evolution and therapy. Cancers (Basel). (2024) 16:3513. doi: 10.3390/cancers16203513, PMID: 39456607 PMC11506062

[25] Wang K Li X Guo S Chen J Lv Y Guo Z. Metabolic reprogramming of glucose: the metabolic basis for the occurrence and development of hepatocellular carcinoma. Front Oncol. (2025) 15:1545086. doi: 10.3389/fonc.2025.1545086, PMID: 39980550 PMC11839411

[26] Yang Y Gao Y Xiong Y Gong Y Lu J Zhang Y. Research progress of warburg effect in hepatocellular carcinoma. Front Biosci (Landmark Ed). (2024) 29:178. doi: 10.31083/j.fbl2905178, PMID: 38812302

[27] Bracker TU Sommer A Fichtner I Faus H Haendler B Hess-Stumpp H. Efficacy of MS-275, a selective inhibitor of class I histone deacetylases, in human colon cancer models. Int J Oncol. (2009) 35:909–20., PMID: 19724929 10.3892/ijo_00000406

[28] Yar Saglam AS Yilmaz A Onen HI Alp E Kayhan H Ekmekci A. HDAC inhibitors, MS-275 and salermide, potentiates the anticancer effect of EF24 in human pancreatic cancer cells. EXCLI J. (2016) 15:246–55., PMID: 27330528 10.17179/excli2016-186 PMC4908665

[29] Pozarowski P Darzynkiewicz Z. Analysis of cell cycle by flow cytometry. Methods Mol Biol. (2004) 281:301–11. 10.1385/1-59259-811-0:301 15220539

[30] Mariotti S Pardini M Teloni R Gagliardi MC Fraziano M Nisini R. A method permissive to fixation and permeabilization for the multiparametric analysis of apoptotic and necrotic cell phenotype by flow cytometry. Cytometry A. (2017) 91:1115–24. doi: 10.1002/cyto.a.23268, PMID: 29072808

[31] Liu X Guo C Leng T Fan Z Mai J Chen J. Differential regulation of H3K9/H3K14 acetylation by small molecules drives neuron-fate-induction of glioma cell. Cell Death Dis. (2023) 14:142. doi: 10.1038/s41419-023-05611-8, PMID: 36805688 PMC9941105

[32] Jeanblanc J Lemoine S Jeanblanc V Alaux-Cantin S Naassila M. The class I-specific HDAC inhibitor MS-275 decreases motivation to consume alcohol and relapse in heavy drinking rats. Int J Neuropsychopharmacol. (2015) 18:pyv029. doi: 10.1093/ijnp/pyv029, PMID: 25762717 PMC4576514

[33] Verrillo L Di Palma R de Bellis A Drongitis D Miano MG. Suberoylanilide hydroxamic acid (SAHA) is a driver molecule of neuroplasticity: implication for neurological diseases. Biomolecules. (2023) 13:1301. doi: 10.3390/biom13091301, PMID: 37759701 PMC10526795

[34] Stacpoole PW Kurtz TL Han Z Langaee T. Role of dichloroacetate in the treatment of genetic mitochondrial diseases. Adv Drug Delivery Rev. (2008) 60:1478–87. doi: 10.1016/j.addr.2008.02.014, PMID: 18647626 PMC3746325

[35] Halasi M Wang M Chavan TS Gaponenko V Hay N Gartel AL. ROS inhibitor N-acetyl-L-cysteine antagonizes the activity of proteasome inhibitors. Biochem J. (2013) 454:201–8. doi: 10.1042/BJ20130282, PMID: 23772801 PMC4322432

[36] Park B Kim JY Riffey OF Dowker-Key P Bruckbauer A McLoughlin J. Pyruvate kinase M1 regulates butyrate metabolism in cancerous colonocytes. Sci Rep. (2022) 12:8771. doi: 10.1038/s41598-022-12827-9, PMID: 35610475 PMC9130307

[37] Khandelwal A Gediya L Njar V. MS-275 synergistically enhances the growth inhibitory effects of RAMBA VN/66–1 in hormone-insensitive PC-3 prostate cancer cells and tumours. Br J Cancer. (2008) 98:1234–43. doi: 10.1038/sj.bjc.6604295, PMID: 18349838 PMC2359640

[38] Du L Wang D Wei X Liu C Xiao Z Qian W. MS275 as Class I HDAC inhibitor displayed therapeutic potential on Malignant ascites by iTRAQ-based quantitative proteomic analysis. BMC Gastroenterol. (2022) 22:29. doi: 10.1186/s12876-022-02101-7, PMID: 35062876 PMC8783488

[39] Yang GC Yang GY Tao LC. Distinguishing well-differentiated hepatocellular carcinoma from benign liver by the physical features of fine-needle aspirates. Mod Pathol. (2004) 17:798–802. doi: 10.1038/modpathol.3800121, PMID: 15044922

[40] Zhu K Xia Y Tian X He Y Zhou J Han R. Characterization and therapeutic perspectives of differentiation-inducing therapy in Malignant tumors. Front Genet. (2023) 14:1271381. doi: 10.3389/fgene.2023.1271381, PMID: 37745860 PMC10514561

[41] Chao J Zhao S Sun H. Dedifferentiation of hepatocellular carcinoma: molecular mechanisms and therapeutic implications. Am J Transl Res. (2020) 12:2099–109., PMID: 32509204 PMC7269980

[42] Shinkawa H Tanaka S Kabata D Takemura S Amano R Kimura K. The prognostic impact of tumor differentiation on recurrence and survival after resection of hepatocellular carcinoma is dependent on tumor size. Liver Cancer. (2021) 10:461–72. doi: 10.1159/000517992, PMID: 34721508 PMC8527909

[43] Xing F Luan Y Cai J Wu S Mai J Gu J. The Anti-Warburg Effect Elicited by the cAMP-PGC1alpha Pathway Drives Differentiation of Glioblastoma Cells into Astrocytes. Cell Rep. (2017) 18:468–81. doi: 10.1016/j.celrep.2016.12.037, PMID: 28076790 PMC5926788

[44] Aggarwal V Tuli HS Varol A Thakral F Yerer MB Sak K. Role of reactive oxygen species in cancer progression: molecular mechanisms and recent advancements. Biomolecules. (2019) 9:735. doi: 10.3390/biom9110735, PMID: 31766246 PMC6920770

[45] de Almeida AJPO de Oliveira JCPL da Silva Pontes LV de Souza Júnior JF Gonçalves TAF Dantas SH. ROS: basic concepts, sources, cellular signaling, and its implications in aging pathways. Oxid Med Cell Longev. (2022) 2022:1225578. doi: 10.1155/2022/1225578, PMID: 36312897 PMC9605829

[46] Sun S Xue D Chen Z Ou-Yang Y Zhang J Mai J. R406 elicits anti-Warburg effect via Syk-dependent and -independent mechanisms to trigger apoptosis in glioma stem cells. Cell Death Dis. (2019) 10:358. doi: 10.1038/s41419-019-1587-0, PMID: 31043589 PMC6494878

[47] Safri F Nguyen R Zerehpooshnesfchi S George J Qiao L. Heterogeneity of hepatocellular carcinoma: from mechanisms to clinical implications. Cancer Gene Ther. (2024) 31:1105–12. doi: 10.1038/s41417-024-00764-w, PMID: 38499648 PMC11327108

[48] Nowak D Stewart D Koeffler HP. Differentiation therapy of leukemia: 3 decades of development. Blood. (2009) 113:3655–65. doi: 10.1182/blood-2009-01-198911, PMID: 19221035 PMC2943835

[49] Llovet JM Ricci S Mazzaferro V Hilgard P Gane E Blanc JF. Sorafenib in advanced hepatocellular carcinoma. N Engl J Med. (2008) 359:378–90. doi: 10.1056/NEJMoa0708857, PMID: 18650514

[50] Cardoso H Alves AM Marques M Vale AM Pereira P Macedo G. Hepatocellular carcinoma treatment with sorafenib: real-life evaluation of prognostic factors and a practical clue for patient management. GE Port J Gastroenterol. (2016) 23:243–8. doi: 10.1016/j.jpge.2016.04.006, PMID: 28868469 PMC5580019

[51] Zhu YJ Zheng B Wang HY Chen L. New knowledge of the mechanisms of sorafenib resistance in liver cancer. Acta Pharmacol Sin. (2017) 38:614–22. doi: 10.1038/aps.2017.5, PMID: 28344323 PMC5457690

