# Supplementary figures and images for: HDAC inhibitor MS275 reprograms metabolism to induce differentiation and suppress proliferation in hepatocellular carcinoma
Source: Front Immunol. 2025 Sep 16;16:1623211. doi: 10.3389/fimmu.2025.1623211 (PMC12479291; doi:10.3389/fimmu.2025.1623211)

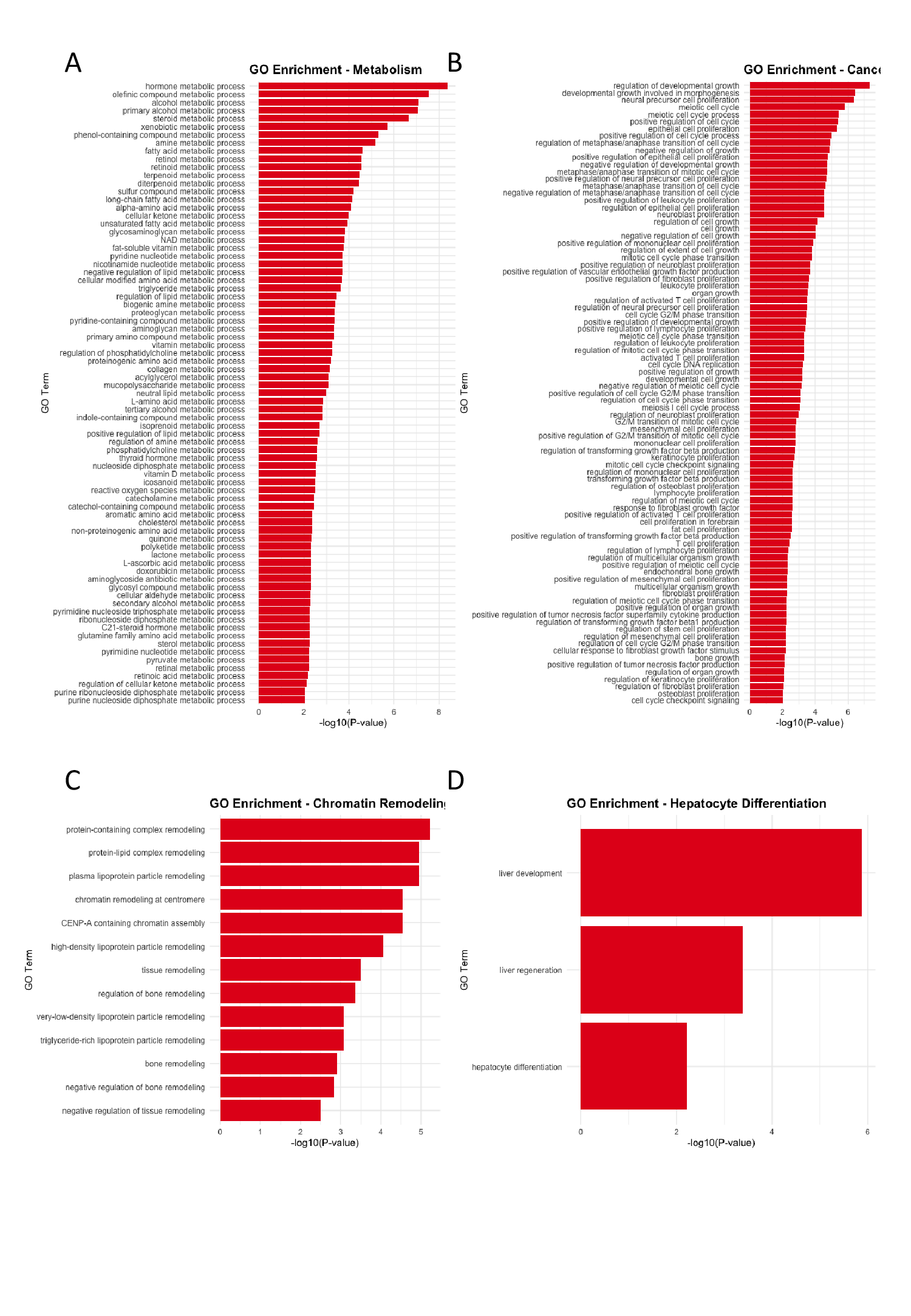

Supplement: Supplementary Figure 1 — GO enrichment analysis highlights key biological processes altered by MS275 treatment. (A-D) GO terms related to metabolism (A), cancer-related processes (B), chromatin remodeling processes (C), and hepatocyte differentiation-related processes (D). [file Image1.tiff]
